# Synthesis of bimetallic nanoparticles loaded on to PNIPAM hybrid microgel and their catalytic activity

**DOI:** 10.1038/s41598-021-94177-6

**Published:** 2021-07-20

**Authors:** Mohib Ullah Kakar, Khakemin Khan, Muhammad Akram, Rokayya Sami, Ebtihal Khojah, Imran Iqbal, Mahmoud Helal, Abdul Hakeem, Yulin Deng, Rongji Dai

**Affiliations:** 1 Beijing Key Laboratory for Separation and Analysis in Biomedicine and Pharmaceutical, Beijing Institute of Technology (BIT), Beijing, 100081 People’s Republic of China; 2 Faculty of Marine Sciences, Lasbela University of Agriculture, Water and Marine Sciences (LUAWMS), Uthal, Balochistan Pakistan; 3 Department of Chemistry, Hazara University, Khyber Pakhtunkhwa, Pakistan; 4 Institute for Synthetic Biosystem, School of Chemistry and Chemical Engineering, Beijing Institute of Technology, Beijing, 100081 People’s Republic of China; 5 Department of Food Science and Nutrition, College of Sciences, Taif University, P.O. 11099, Taif, 21944 Saudi Arabia; 6 Department of Information and Computational Sciences, School of Mathematical Sciences and LMAM, Peking University, Beijing, 100871 People’s Republic of China; 7 Department of Mechanical Engineering, Faculty of Engineering, Taif University, Taif, Saudi Arabia

**Keywords:** Chemistry, Materials science

## Abstract

This study was designed to preparecarboxyl-functionalized poly (N-isopropylacrylamide) PNIPAM microgels having excellent catalytic properties.Recently, researchers are trying to fabricate cost effective and efficient hybrid catalytic materials for the synthesis of nitrogenous compounds along with enhanced optical properties. For the same motive, synthesis of carboxyl-functionalized PNIPAM microgels was performed by using polymerization of soap-free emulsion of N-isopropyl acrylamide, which is NIPAM along with acrylic acid (AA). The thiol group was introduced through the imide bond mediated by carbodiimide, between carboxyl-functionalized microgels through carboxyl group and aminoethanethiol (AET). Copper, Palladium and Cu/Pd nanoparticles were incorporated successfully into thiol-functionalized PNIPAM microgels through metals thiol linkage. The synthesized microgels and hybrid encompassing metallic nanoparticles were characterized in detail by using Transmission electron microscopy (TEM), Scanning electron microscopy (SEM), X-ray diffraction (XRD), X-ray photoelectron (XPS) and Fourier transformed infrared spectroscopy for structural interpretation. The thermal properties of the pure and hybrid microgels were inspected by TG analysis. The prepared nanocomposites PNIPAM-Cu, PNIPAM-Pd and PNIPAM-Cu/Pd exhibited decent catalytic properties for the degradation of 4-Nitrophenol and methylene blue, but the bimetallic Cu/Pd have remarkable catalytic properties. The catalytic reaction followed pseudo-first-order reaction with rate constants 0.223 min^−1^, 0.173 min^−1^ for 4-Nitrophenol and methylene blue in that order. In this study,we were able to establish that Cu/Pd hybrid is an efficient catalyst for 4-Nitrophenol and methylene blue as compared to its atomic analogue.

## Introduction

Metallic nanoparticles have charmed attraction for few decades owing to their abundance and variety of applications in catalysis^1^, medicine^2^, bio/imaging^3^, sensors^4^, and electronics^5^. Due to their high surface area and greater surface energy, nanoparticles (NPs) showed prominent catalytic activities like CO oxidation^6^ for nucleation of carbon nanotubes (CNTs)^7^, dehydrogenation of alcohol^8^ as well as electro-oxidation of formic acids^9^. Due to the high surface energy and van der Waal forces, NPs can aggregate, which reduces the surface area as well as catalytic activities. Therefore, metal NPs can be stabilized by using suitable additives like hydrogels^10^, dendrimers^11^, micelles of block copolymers^12^ and gutta-percha particles^13^.

For the specific characteristic properties of nanocomposites, nanoparticles such as carbon-based^14^, ceramic^15^, and metallic nanoparticles^16^ can be incorporated/implanted within the hydrogel network^10^. Nanocomposite hydrogels or hybrid hydrogels (highly hydrated polymeric networks) are either physically or covalently cross-linked with nanoparticles^17–19^. Nanocomposite hydrogels can be constructed in the presence of microgel stabilizers with enhanced physical, chemical, electrical, and biological properties like the hybridization of organic and inorganic nanoparticles including surface Plasmon resonance (SPR) and photoluminescence, etc^20–22^. For the in-situ synthesis of nanocomposite microgel and NPs, hydrogels are primary carrier due to their hydrating ability for catalytic reactions as well as recycling ability without losing the NPs. The functional groups of hydrogel networks like –SO_3_H, –SH, –OH, –NH_2_, and –COOH attract metal ions with electrostatic interactions. It helps to stabilize and reduce the hydrogel through a suitable reducing agent. Therefore, hydrogels are used as real reactors for the in-situ synthesis of metal NPs to avoid aggregation. In recent years, polymer hydrogels have been investigated as an efficient and real reactor for in-situ synthesis of metallic NPs for the decomposition of various aromatic pollutants and dyes^23–25^. Most of the researchers have focused on metallic, nonmetallic and semiconductor nanoparticles, e.g. Au^26^, Ag^27^, Pd^28^, Co^29^, ZnO^30^, CeO_2_^31^, TiO_2_^32^ etc. It is reported in most of the literature that Au, Ag and Pd nanoparticles were used to reduce 4-NP to 4-aminophenol^33–36^. The catalytic activities of gold, silver and palladium nanoparticles, impregnated in microgels, were studied^37–39^.

Bimetallic nanoparticles of two different metals have become very popular in recent years due to their unique and enhanced catalytic properties as compared to their monometallic analogues^40–42^. The catalytic properties of Pd in any bimetallic catalysts are greater as compared to other bimetallic except Pd as one of its components^26,43,44^. Therefore, Pd based bimetallic catalysts are considered as promising candidates for the degradation of different organic and environmental pollutants. For example, Pd/Au^45^, Pd/Ni^46^, Pd/Pt^44^ and Pd/Cu^47^ are investigated as prominent bimetallic materials for various applications.

In this study, we have reported thiol-functionalized microgels to stabilize Cu, Pd, and Cu/Pd bimetallic NPs and applied them to degrade 4-Nitrophenol and methylene blue in which the bimetallic NPs showed efficient catalytic properties. Therefore, we suggest, Cu/Pd bimetallic based microgels are promising catalysts for the degradation of environmental pollutants.

## Experimental

### Materials

N-Isopropyl acrylamide (NIPAM) purchased from Sigma Aldrich, Inc. and purified by recrystallization. For recrystallization purpose, n-hexane was used as a solvent and material was vacuum dried. Reduced nitrogen pressure was used for refinement of acrylic acid (AA). Then deionized water was used for recrystallisation and distillation of Potassium per sulfate (KPS). *N*,*N*-Methylene bisacrylamide (MBA, Alfa Aesar), 1-ethyl-3-(3-dimethylaminopropyl) carbodiimide hydrochloride (EDAC, Alfa Aesar, 98%), Copper nitrate trihydrate Cu (NO_3_)_2_·3H_2_O and PdCl_2_. 2-aminoethanethiol (AET, RED chemical), ascorbic acid and Hydrazine (98.0%) were used as received. All experiments were performed by using double distilled water.

### Carboxyl-functionalized PNIPAM microgels synthesis

We synthesized carboxyl-functionalized microgels through soap-free emulsion polymerization technique by mixing N-Isopropyl acrylamide (NIPAM) and acrylic acids. For this purpose, round bottom flask (three necked, 500 ml capacity) was used having a mechanical stirrer equipped with a nitrogen inlet. The composition of the microgels was as 2 g NIPAM, 0.2 g AA monomer, a cross linker MBA (0.2 g) and KPS as an initiator (0.1 g) with deionized water (300 mL) was stirred with (300 rpm) and continuous supply of nitrogen gas for 30 min. Polymerization was induced by increasing the temperature up to 70 °C, time spanning over 6 h. Purification of these prepared microgels was done by dialysis using the deionized water for 3 days. The water was replaced frequently after 10 h.

### Preparation of thiol-functionalized PNIPAM microgels

Thiol-functionalized PNIPAM microgels were prepared. This preparation was based on the formation of amid bond between the functional group of microgel and amine group of AET. 100 mL of carboxyl-functionalized PNIPAM microgel aqueous dispersion and 0.2 mL AET aqueous solution (20 mL) were charged into three-necked round bottom flask (250 mL capacity) and agitated at (200 rpm) at 20 °C for 10 min. Then, 20 mL of 0.46 M EDAC aqueous solution was added and the pH was adjusted to 6.1 by using 0.2 M aqueous HCl. This reaction performed at 20 °C for 2 h under constant stirring. Excess amounts of unreacted chemicals AET were removed, and the obtained thiol-functionalized PNIPAM microgels dispersion were further dialyzed against deionized water for purification.

### In situ synthesis of metals nanoparticles in PNIPAM microgels

In-situ synthesis of metal nanoparticles in microgels network, 10 mL of 50 ppm metal salts Cu (NO_3_)_2_·3H_2_O, PdCl_2_ was added to 10 mL already prepared microgels which were further diluted by adding 15 mL deionized water respectively. With constant stirring and refluxing at 120 °C, 0.5 mL ascorbic acid (0.05 M) was added. Then 2 mL of NaOH (0.6 M) was added to the solution after further stirring for 20 min. Finally, a 0.5 mL volume (0.05 M) of N_2_H_4_was added and the color of the colloidal solution changed which indicated the formation of metals hybrid nanoparticles.

### Preparation of PNIPAM/Bimetallic Cu/Pd Hybrid microgels

10 mL of (0.05 M) PdCl_2_ was used to prepare the Pd nanoparticles, as described by Turkevich^57^. According to this method, 10 mL of (0.05 M) PdCl_2_ was added to 10 mL of prepared microgels which were further diluted by adding 15 mL of deionized water. 0⋅5 mL ascorbic acid was added after stirring for 20 min and then 10 mL Cu (NO_3_)_2_·3H_2_O (0.05 M) was added. 2 mL NaOH (0.6 M) was added drop wise and we adjusted the pH of the above colloidal solution to 8.5 with continuous stirring for further 30 min. In the end, 0⋅5 mL volume (0⋅05 M) of N_2_H_4_was added and the color of colloidal solution became dark muddy which showed the formation of bimetallic hybrid nanoparticles.

### 4-Nitrophenol and methylene blue reduction, catalyzed by PNIPAM-K hybrid microgels

Thermally tunable catalytic property of bimetallic based PNIPAM microgels was explored 4-Nitrophenol (4-NP) along with methylene blue reduction in the presence of NaBH4 at different range of temperatures. The process was done into a quartz cell for UV–Vis spectroscopy. 1 mL of 4-NP and 1 mL methylene blue aqueous solution (0.1 mM) was separately mixed with 2 mL of 20 mM NaBH_4_ aqueous solution, then added into a quartz cell and test it at given temperature. All at once, the PNIPAM/Cu and PNIPAM/Pd hybrid microgels latex were put in a water bath for acquiring the testing temperature. Afterwards, 0.5 mL of PNIPAM/Cu and Pd latex (0.06 mg/mL) was placed in thee quartz cell and then UV–Vis spectra was obtained after each set interval of time. The reduction rate constant for 4-Nitrophenol was determined by computing the decrease in intensity at 400 nm and for Methylene blue at 650 nm by various catalytic agents. A calibration curve was formed at 400 nm for 4-Nitrophenol and 650 for methylene blue to find the concentration of the reagents. The reduction rates of 4-Nitrophenol and methylene blue were calculated at four different temperatures i.e. 30 °C, 32 °C, 34 °C and 36 °C. During this study, every obtained data was repeated three times for the sake of accuracy.

### Catalytic properties of PNIPAM based bimetallic Cu/Pd by reducing 4-Nitrophenol and methylene blue

Similarly, the thermal tunable catalytic properties of PNIPAM based bimetallic copper and palladium alloy were studied by reducing 4-Nitrophenol and methylene blue at a given range of temperature. One mL of each methylene blue and 4-Nitrophenol, 0.1 mM aqueous solution separately mixed with 10 mM NaBH_4_ aqueous solution (1 mL). The solution was then adjusted to the given temperature. Along with this, the PNIPAM-Cu/Pd hybrid microgels were placed for 10 min in the water bath for acquiring the testing temperature. Later on, 0.2 mL of PNIPAM-Cu/Pd hybrid microgels latex (0.06 mg/mL) was also added into the quartz cell, and then at each set time interval, UV–Vis spectra was taken without any delay. . The reduction rate constant for nitrophenol was determined by computing the decrease in intensity at 400 nm and for methylene blue at 650 nm by various catalytic agents. A calibration curve was formed at 400 nm for 4-Nitrophenol and 650 nm for methylene blue in order to find the concentration of the reagents. The rate of reduction of 4-Nitrophenol and methylene blue were calculated at four different temperatures i.e. 30 °C, 32 °C, 34 °C and 36 °C. During this study, every obtained data was repeated three times for the sake of accuracy.

### Characterization

#### Fourier transform infrared spectroscopy

Fourier transform infrared spectroscopy was performed on a Perkin Elmer Spectrum BX Fourier transform Infrared spectrometer (Perkin Elmer, Waltham, MA, USA). The dried samples of microgels were analyzed by observing various peaks for the different functional groups.

#### UV–visible spectroscopy

UV–visible spectroscopy was performed by using at a Shimadzu UV-2450 spectrometer was used for testing the UV–Vis spectroscopy. A temperature controller (TCC-240A) was equipped with it.

#### Thermo gravimetric analysis

By using a Shimadzu DTG-60H instrument, we analyzed all samples under nitrogen atmosphere (50 mL/min) at the heating rate of 10 °C/min.

#### Transmission electron microscopy (TEM)

The morphology of the pure microgel and hybrid gels were studied by using transmission electron microscopy (TEM). For TEM sampling, microgels were diluted by distilled and then a sample drop was put on a stander copper grid supported by carbon film. The samples were dried at 50 °C and characterized by TEM with an accelerating voltage of 100 kV.

#### X-rays diffraction (XRD)

The phase purity of Bimetallic nanoparticles was analyzed by X-ray diffraction (XRD) using a Bruker D2 PHASER X-ray Diffractometer with a graphite monochromator using Cu Kα radiation (λ = 1.54056 Å), operating at 30 kV and 15 mA.

#### X-rays photoelectron spectroscopy (XPS)

The X-ray photoelectron spectroscopy (XPS) was performed on VG ESCALAB MKII spectrometer equipped with Al-Kα X-ray source.

#### Scanning electron microscopy (SEM)

For SEM characterization, the samples were dropped on the silicon single crystal sheet, dried by IR light, which was carefully placed on conducting glue. Then all the samples were coated with gold vapor in order to become conducting and analyzed by JSM 6700F SEM.

### Consent to publish

All co-authors are willing to publish the data in this journal.

## Results and discussion

**Figure 1:**
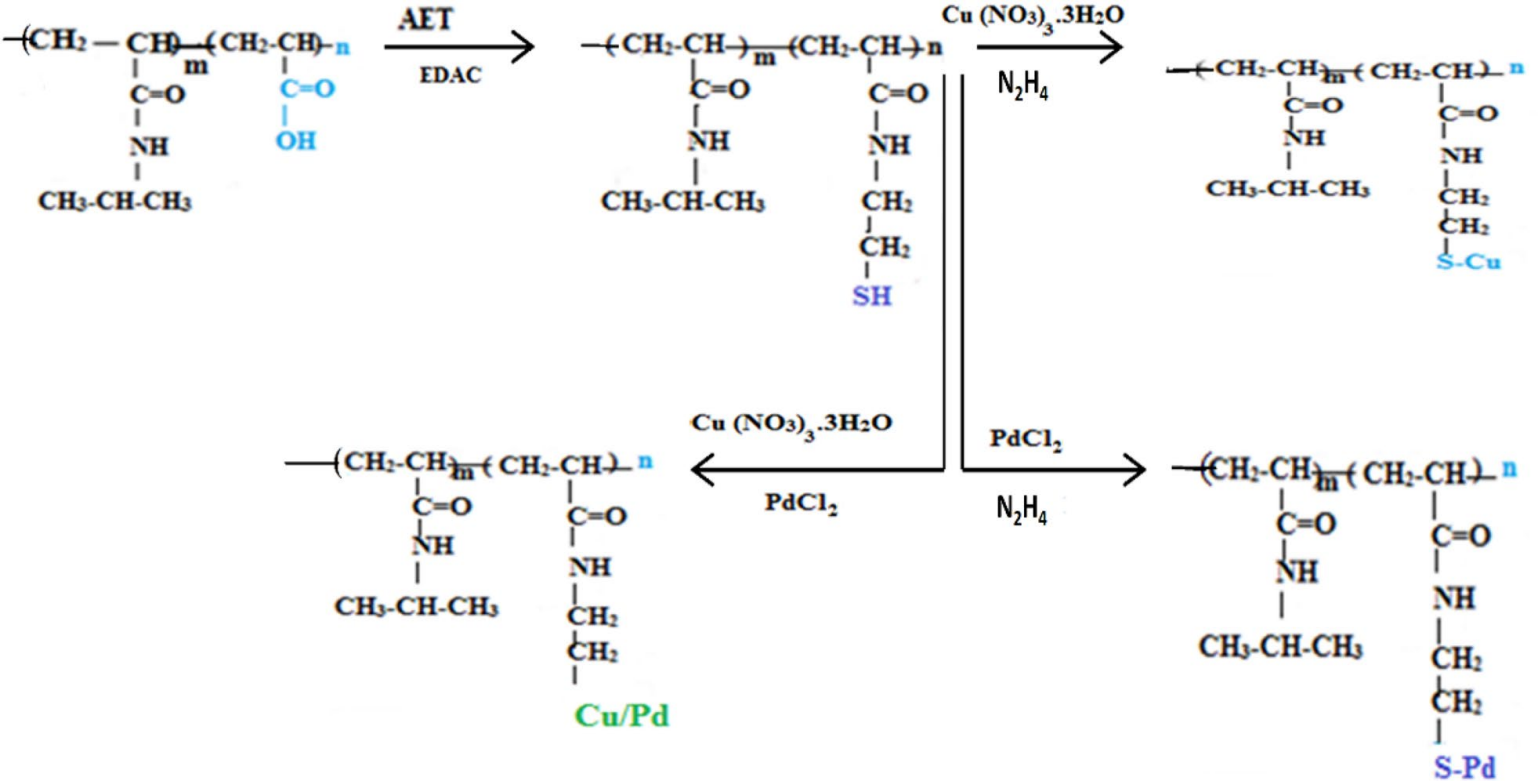
Schematic representation for the preparation of PNIPAM–K hybrid microgel.

In this study, we were efficiently able to synthesis thio-functionalized poly N-isopropyl acrylamide microgels, which were impregnated by Copper Palladium and Cu/Pd nanoparticles successfully, as shown in Fig. 1. The carboxyl functionalized PNIPAM microgels were first prepared by soap-free emulsion polymerization. In polymerization, NIPAM acts as a temperature-sensitive monomer and acrylic acid used as a functional monomer. Then thiol group was introduced in the PNIPAM microgels by carbodiimide mediated reaction between the carboxyl group and 2-aminethanethiol (AET). The NPs of Cu, Pd and Cu/Pd Bimetallic were impregnated on thiol functionalized PNIPAM microgels particles. The color of the reaction mixture transformed instantaneously by the addition of reducing agent that indicated the formation of metallic nanoparticles (MNPs) into the microgels network. In-situ*,* the metallic NPs*,* were synthesized by the reduction of metal salt precursors having thermally tunable optical and excellent catalytic properties. The morphologies of pure microgel and Copper, Palladium based hybrid microgels were obtained by TEM, while the bi-metallic hybrid microgels were investigated by SEM, XRD and XPS.

**Figure 2:**
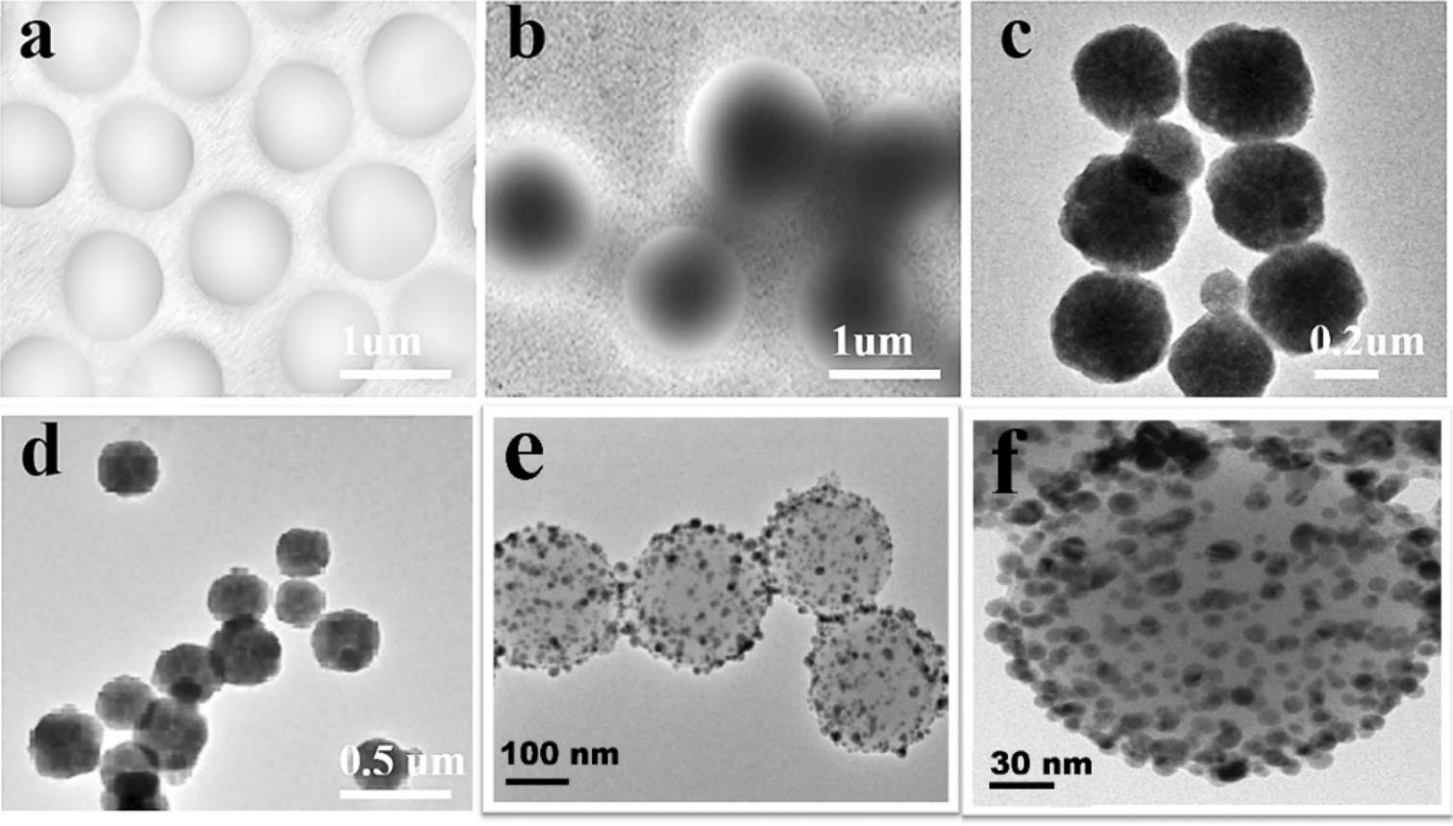
TEM images of (**a**) carboxyl-functionalized PNIPAM microgels, (**b**) thiol-functionalized PNIPAM microgels, (**c**) PNIPAM–Cu hybrid microgels (**d**) PNIPAM–Pd hybrid microgels and (**e**, **f**) Hexagonal core–shell nanoparticles of Cu/Pd.

The TEM photographs of the hybrid microgels showed that copper and palladium nanoparticles are uniformly distributed in the PNIPAM microgels and not aggregated, as shown in Fig. 2c,d. The average diameter of the Cu NPs is 6 ± 1 nm and that Pd NPs is 5 ± 1 nm. Similarly, the core–shell nanoparticles of Cu/Pd are visualized by SEM which shows hexagonal shapes as shown in Fig. 2.

**Figure 3:**
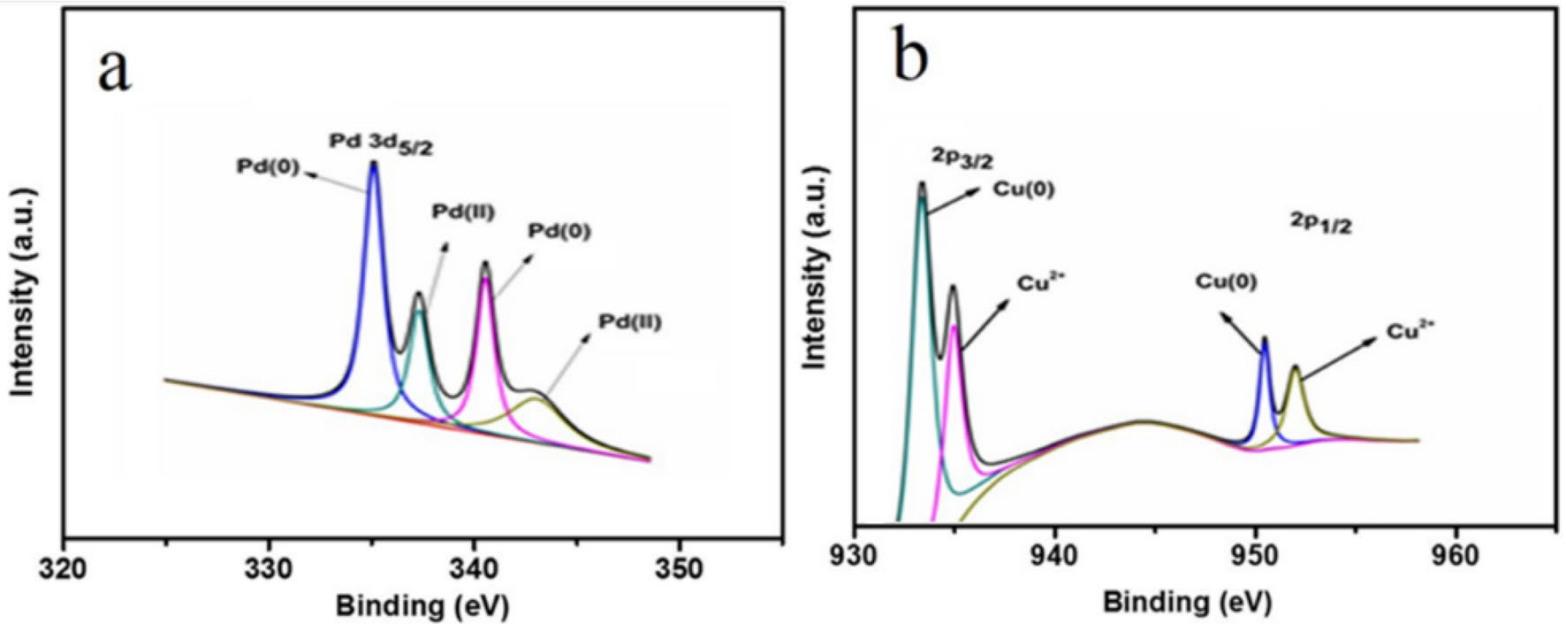
(**a**) XPS Spectra of Pd and (**b**) XPS spectra of copper. (OriginPro 2019b (64-bit) 9.6.5.169 version) https://downloadly.ir/software/engineering-specialized/originpro/.

To determine the oxidation states and surface compositions of the as prepared samples, X-ray photoelectron spectroscopy (XPS) characterization technique was employed. The Pd 3d and Cu 2p regions of the spectrum are given in Fig. 3a,b. Origin Pro 2019b software was used for XPS peaks fitting. Gaussian–Lorentzian function was employed for the peak fitting and Shirley type background correction was applied.

The binding energy in the range of 335–343 eV relating to the Pd 3d5/2 and 3d3/2 were observed in XPS spectra. The two intensive peaks as shown in Fig. 3a at 334.6 and 340.9 eV, confirmed the presence of metallic palladium (0). While the other doublets at 337.9 and 343.4 eV shows that palladium existence in its divalent state^48,49^.

In the case of copper, it is challenging to differentiate Cu_2_O from metallic Cu in the Cu 2p region of the XPS spectrum^50^. Although, the binding energies for Cu 2p3/2 were investigated in the XPS spectra at 932.2 eV and 934.6 eV, which confirmed the existence of copper in its both oxidation states (zero oxidation Cu (0) and divalent state Cu (II))^9^. Likewise, the binding energies for Cu 2p1/2 were also observed in the spectra at 951.0 and 953.1, which are representing Cu (0) and Cu (II), respectively as shown in Fig. 3b^50,51^. It can be seen that the oxidation states of Cu (0) are dominant than the Cu (II) values, which confirmed the presence of Cu in its metallic state^49^. Consequently, these findings confirmed that the palladium and copper nanoparticles are physically interacted with the surface of the hydrogel matrix.

**Figure 4:**
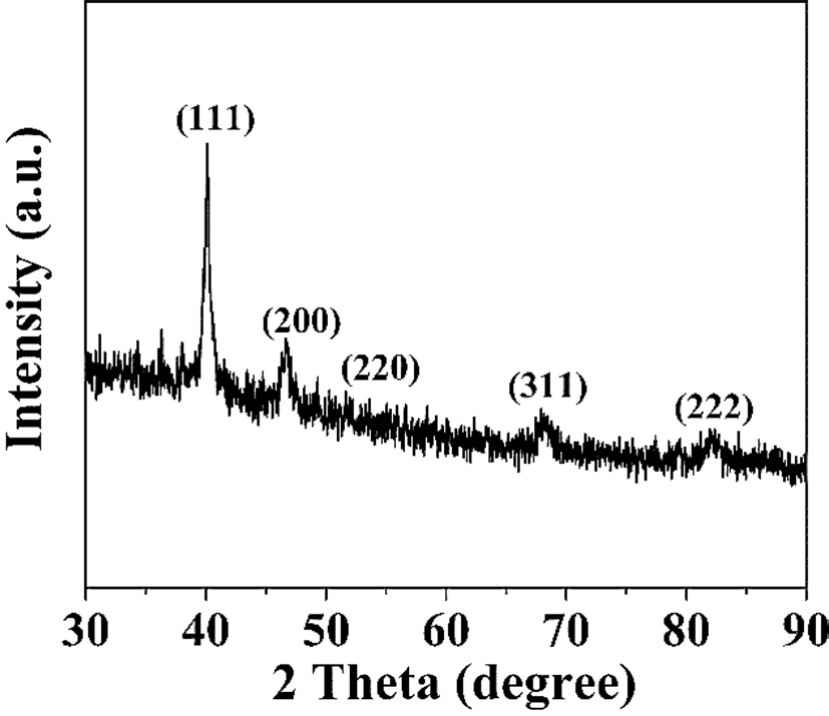
XRD Pattern for Copper palladium nanocrystal.

The XRD studies of Cu/Pd hexagonal crystals are shown in Fig. 4. The diffracted peaks observed at 2θ value of 41°, 48°, 70°, 85°, and 90° correspond to the lattice plane (111), (200), (220), (311), and (222) respectively of CuPd (JCPDS card no. 48-1551) which shows resemblance with the SAED pattern as shown in Fig. 4.

**Figure 5:**
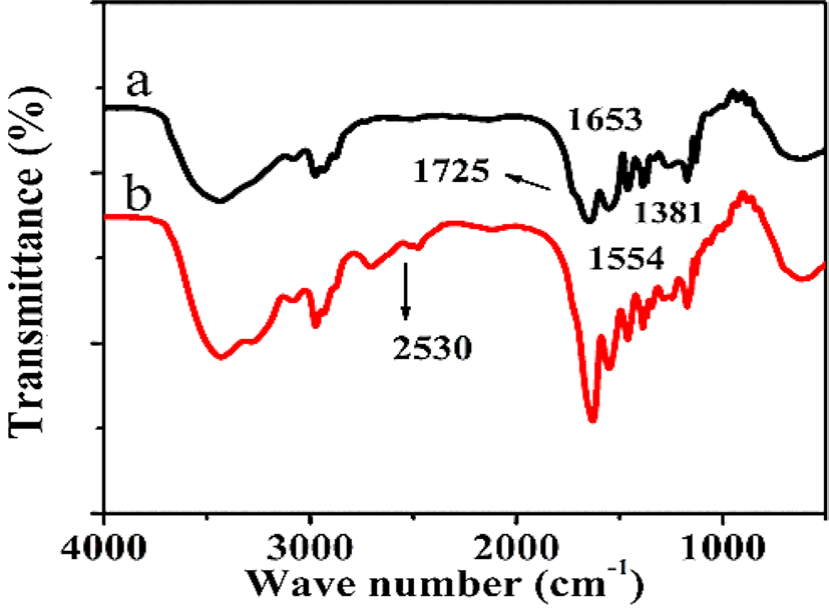
FT-IR spectra of (**a**) carboxyl-functionalized PNIPAM, and (**b**) thiol-functionalized PNIPAM microgels.

FT-IR spectrum shown in Fig. 5 signifies the absorption peaks for amide I band at 1653, 1554, and 1381 cm^−1^ which is due to C=O stretching vibration^52,53^. The bending vibration of N–H, CH_3_ and stretching vibration of C–N are collectively assigned to amide II band. The small peak shoulder at 1725 cm^−1^ represents the stretching vibration of C=O of the AA which produces a very dynamic o-acylisourea intermediate and reacts with strong nucleophile like primary amine to form amide bond. AET in the above reaction is used as a nucleophile to produce thiol group in the PNIPAM microgels. In the FT-IR spectrum for thiol-functionalized PNIPAM microgels (Fig. 5), the shoulder of the peak at 1725 cm^−1^ is very weak when compared with the carboxyl functionalized PNIPAM microgels (Fig. 5). Along with this, another weak peak at 2530 cm^−1^ represents the stretching band of S–H and a successful introduction of thiol group in the PNIPAM microgels^53,54^. In addition, the peaks at 1554 cm^−1^and 1653 are both characteristic peaks of PNIPAM.

Thermo gravimetric analysis was performed to measure the thermal properties of hybrid microgels. We have only performed TG of thiol functionalized and copper hybrid microgels and compared the thermo gram of pure and copper hybrid microgels. It shows the difference between the degradation profile of pure and metals encapsulated hybrid microgels from 30 to 600 °C. The weight of the PNIPAM-Cu hybrid microgels remained 55% of the original weight upon elevating the temperature up to 600 °C as shown in supporting information data Fig. S3. The TGA curve demonstrates the thermal stability of the hybrid microgels as compared to the thermo grams of the pure microgels. It is due to the encapsulation of metallic NPS in the microgels network. But the exact amount of the nanoparticles cannot be determined from it due to the formation of metallic oxides.

### Catalytic performance of PNIPAM-Cu hybrid microgels

The catalytic activities of PNIPAM-Cu, PNIPAM-Pd and PNIPAM- Cu/Pd Bimetallic were probed by the reduction of 4-Nitrophenol (4-NP), and methylene blue with an excess volume of NaBH_4_ as a sample reaction. For catalytic activity reactions, nitro compounds i.e. 4-Nitrophenol and dye methylene blue were used. The nitro compounds are very hazardous for health and are widely used in pharmaceutical products and dyes as well as pigments industries. A variety of pharmaceutical products like analgesics and antipyretics are obtained from nitro compounds. Excessive waste of nitro compounds causes many health and environmental issues^29,42^. The technique used for the reduction of these toxic compounds was UV–visible spectroscopy. It was experienced that aqueous solution of sodium borohydride gives recovered rate of reduction with catalyst. The rate of reduction is insignificant without catalyst due to the large potential difference between electron donor and acceptor species in excess reducing agents. So, the catalyst plays an imperative role to reduce the energy barrier in order to make it possible to reduce these products.

**Figure 6:**
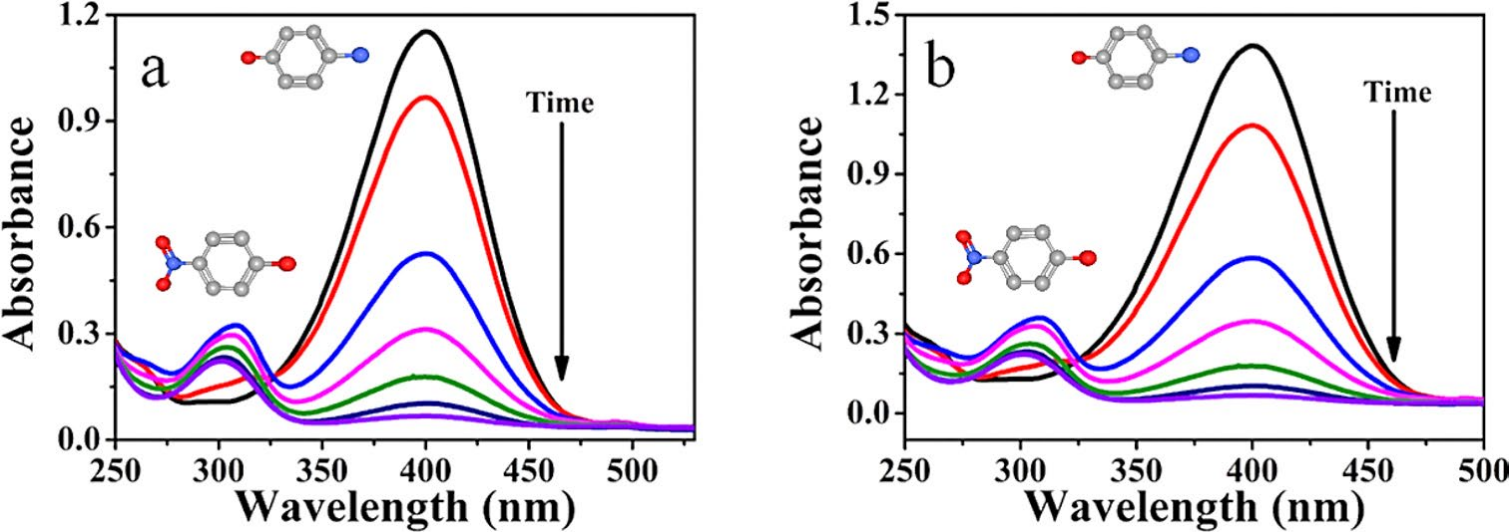
(**a**) Stepwise UV–Vis spectra of the reduction of 4-Nitrophenol catalyzed by NIPAM-Cu hybrid Microgels (**b**) by PNIPAM-Cu/Pd hybrid microgels.

The reduction of 4-Nitrophenol and methylene blue were carried out in the presence of an excess amount of NaBH_4_. The catalysts monometallic Cu, Pd and bimetallic Cu/Pd Hybrid accelerate the reduction reaction by transferring an electron from electron donor species BH_4_^–1^ toelectron acceptor species i.e. 4-Nitrophenol. By using the catalyst, the 4-Nitrophenol was reduced by H ions of NaBH4 and converted into the 4-aminophenol, which is less toxic as compared with the 4-Nitrophenol. The rate of reduction of 4-Nitrophenol was monitored through UV–visible spectroscopy by calculating the decrease in the absorbance at 400 nm as given in Fig. 6, while on the same time the new peaks are prominent at 300 nm, which confirmed the presence of 4-aminophenol.

**Figure 7:**
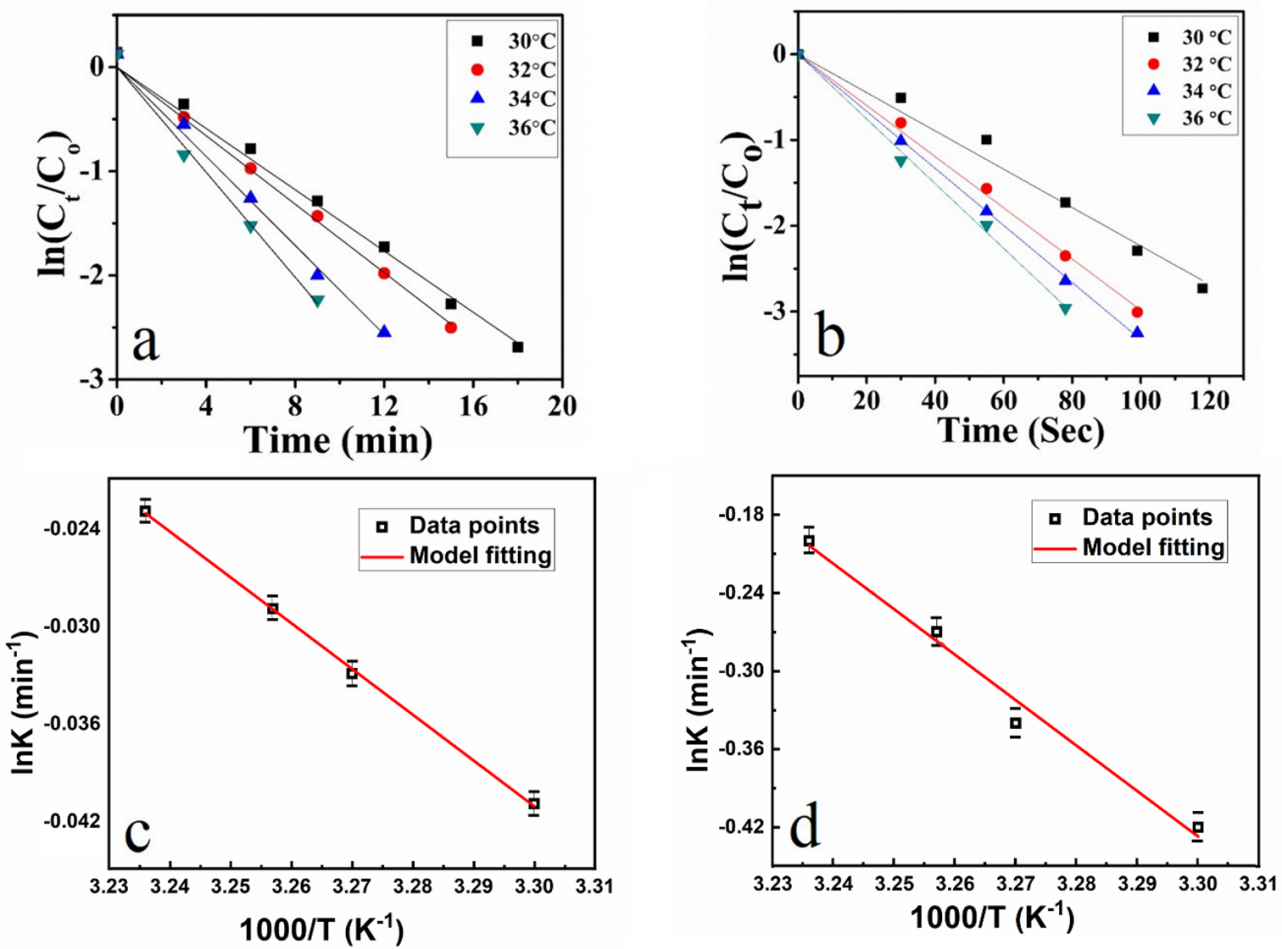
(**a**, **b**) Plot of ln (C_t_/C_0_) versus reaction time at a temperature of 30–36 °C for PNIPAM-Cu and PNIPAM-Cu/Pd respectively, where C_t_ is the concentration of 4-NP at time t and C_0_ is the concentration of 4-NP at t = 0. (**c**) Plot of K_app_ versus temperature in the presence of Cu hybrid (**d**) plot of K_app_ versus temperature in the presence of bimetallic.

The rate of reduction of 4-Nitrophenol was studied at four different temperatures, i.e. 30 °C, 32 °C, 34 °C, 36 °C as shown in Fig. 7a. The kinetics of photo catalytic degradation of dyes can be depicted by first-order equation, which is given as follows;

$${\text{ln}}\left(\frac{{C}_{t}}{{C}_{0}}\right)= {K}_{app}t$$

where K_app_, C_o_ and C_t_ are the pseudo-first-order rate constant (min^−1^), initial concentration of the dye, the concentration of dye solution at time *t* (min). The pseudo-first-order rate constant calculated from the slope of ln (C_t_/C_0_) vs time plot for different catalysts were found to be 0.11571.

The ln (C_t_/C_0_) determined from the UV–Vis spectra is plotted as a function of reaction time which determines "ln (C_t_/C_0_)" as shown in Fig. 7a,b. The plot of ln (C_t_/C_0_) versus time for reduction reaction indicates pseudo first-order kinetics. The slope of the curve at different temperatures shows that apparent rate constant (K_app_) increases with increase in temperature, as shown in Fig. 7c,d. Increase in temperature increases the average kinetic energy and effective collisions of reactants (4-Nitrophenol, NaBH_4_, microgels). So, the reduction rate of 4-Nitrophenol increased with increase in temperature.

The factors like temperature, nature and composition of metal hybrid on the rate of reduction of 4-Nitrophenol have been studied. It was observed by UV/Vis spectroscopy that bimetallic Cu/Pd hybrid microgels give a higher rate of reduction as compared to its atomic analogue as shown in Fig. 6b. The effect of temperature on the rate reduction of 4-Nitrophenol by bimetallic nanohybrid was also studied at four different temperatures i.e. 30 °C, 32 °C, 34 °C, 36 °C as shown in Fig. 7b. The rate of reduction of 4-Nitrophenol by PNIPAM/Pd hybrid microgels is nearly the same as PNIPAM/Cu hybrid microgels, as shown in Supplementary Fig. S1. The apparent rate constant from the slope of the curve at various temperatures followed pseudo first-order kinetics as shown in Fig. 7d. The reduction rate constant obtained from ln (C_t_/C_0_) vs time for 4-Nitrophenole at 30 °C by Cu and bimetallic Cu/Pd hybrid nanocomposites were 0.0223 min^−1^ and 0.223 min^−1^ respectively.

The reduction rate constant of 4-Nitrophenol by using bimetallic Cu/Pd nanocomposite is exceptionally greater than the rate of reduction of 4-Nitrophenol determined by other researchers reported in literature as shown in Table 1.

**Table 1:** Comparison of the PNIPAM-Cu/Pd catalyst with the literature reported catalysts for the 4-NP reduction.

Nano catalyst	Kapp (min^−1^)	References
PNIPAM-Pd	0.082	^54^
AuNP/T-G hydrogel	0.0022	^55^
PVP-Pd	0.086	^54^
PNIPAM-AgNS	0.159	^53^
CN-supported PdNPs nanohybrids	9.5 × 10–5	^56^
MXene@PdNPs	0.003	^57^
Polyethyleneimine (PEI)/Polycaprolactone (PCL)@PdNPs	0.00276	^58^
Pd–Cu bimetallic nanomaterials (BNMs)	0.026	^43^
PNIPAM-Cu/Pd	0.223	**This work**

**Figure 8:**
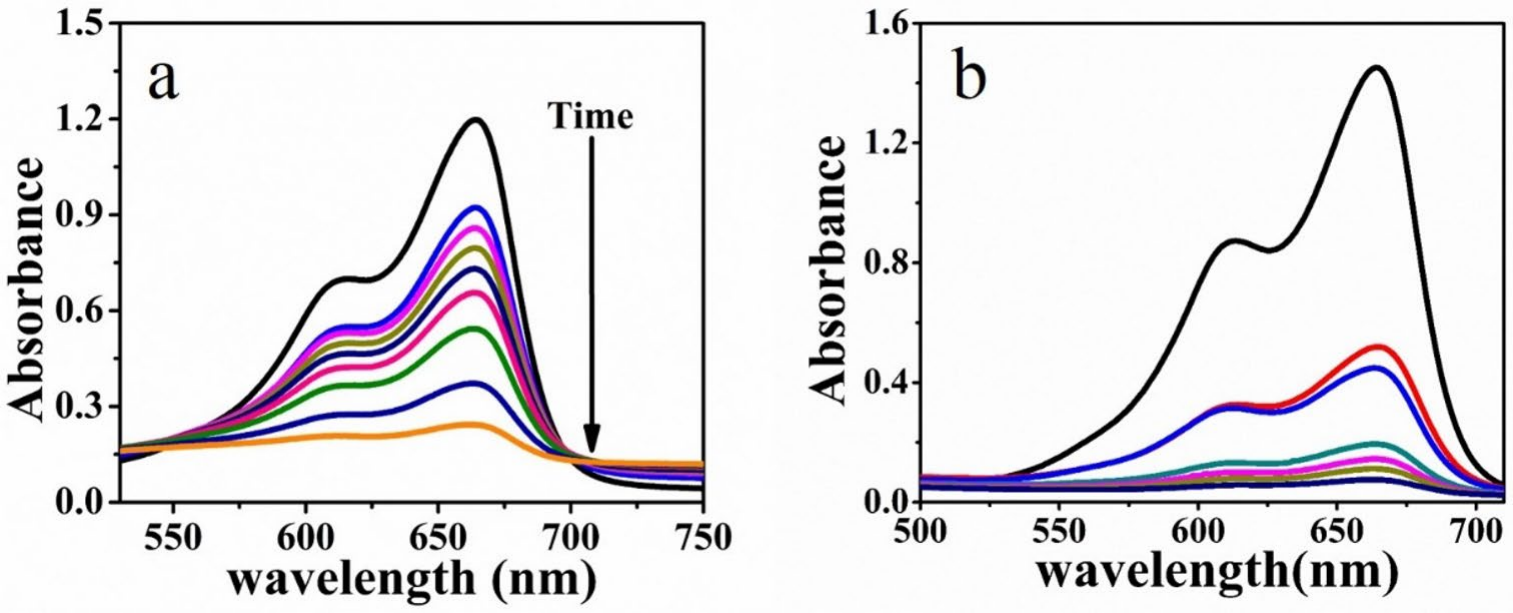
(**a**) Stepwise UV–Vis spectra of the reduction of MB catalyzed by PNIPAM-Cu hybrid microgels (**b**) by PNIPAM-Cu-Pd hybrid microgels.

**Figure 9:**
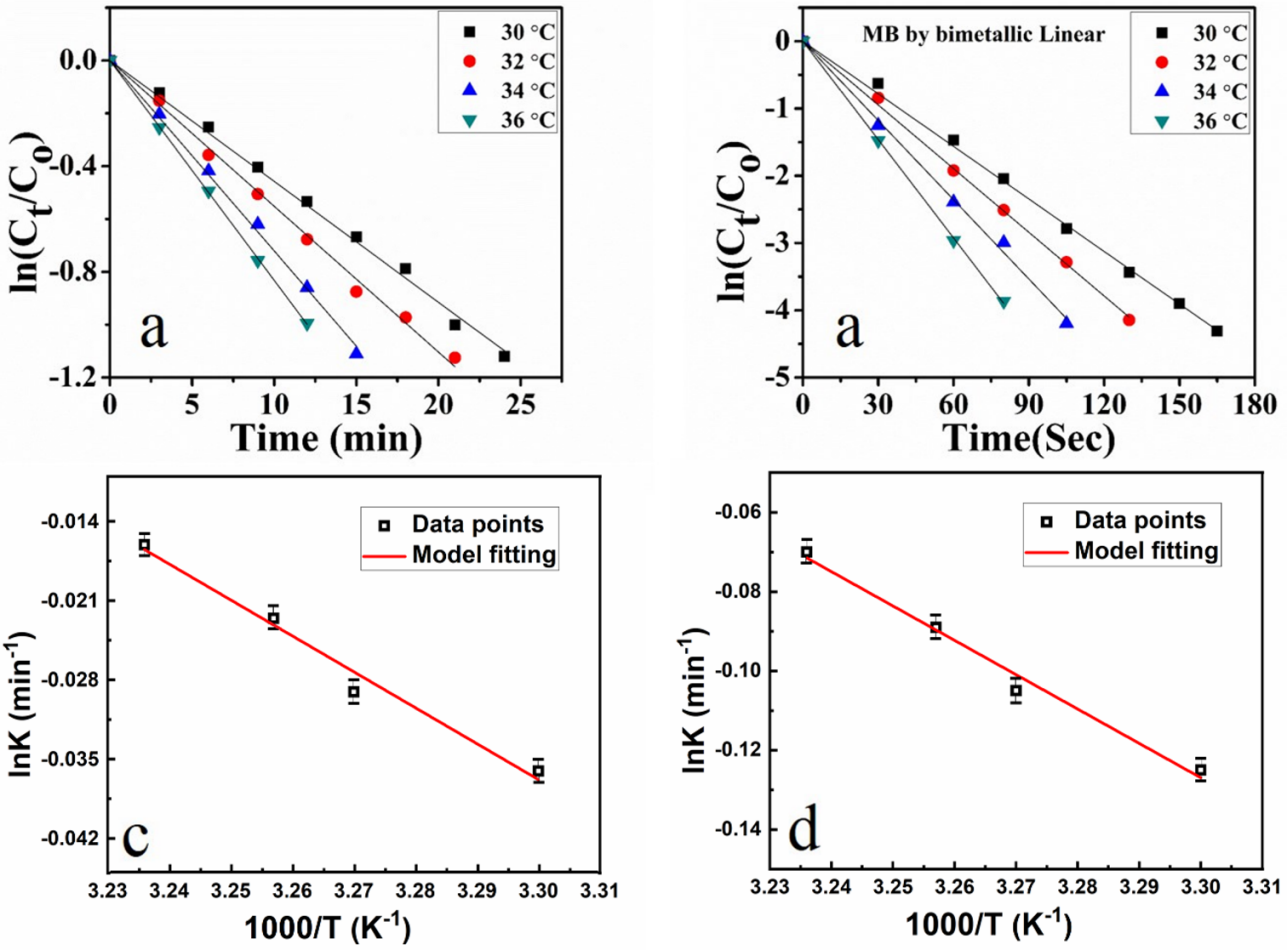
(**a**, **b**) Plot of ln (C_t_/C_0_) versus reaction time at a temperature of 30–36 °C for PNIPAM-Cu and PNIPAM-Cu/Pd respectively, where C_t_ is the concentration of MB at time t and C_0_ is the concentration of MB at t = 0. (**c**, **d**) Plot of lnkapp versus temperature for Cu hybrid and bimetallic nanocomposites respectively.

Correspondingly, the catalytic activities of PNIPAM-Cu/Pd, PNIPAM-Cu, and PNIPAM-Pd hybrid microgels were also investigated through the reduction of methylene blue in the presence of sufficient amount of NaBH_4_ as a reducing agent at various temperatures (30–36 °C). The absorption peak of MB at 650 nm gradually reduced by the addition of PNIPAM-M catalysts, like PNIPAM-Cu/Pd, PNIPAM-Cu and PNIPAM-Pd hybrid microgels as shown in Fig. 8. From UV–visible spectra, ln (C_t_/C_0_) was calculated and plotted as a function of reaction time as shown in Fig. 9a,b and Fig. S2. This plot shows a linear relationship, which proves that the reduction reaction follows pseudo first-order kinetics (Fig. 8).

The apparent rate constants (Kapp) were calculated at various temperatures for all hybrid microgels. The Kapp increases with increase in temperature as observed from the slope of the curve for Cu hybrid and bimetallic Cu/Pd nanocomposites as shown in Fig. 9c,d. These catalytic activities are due to two factors i.e. diffusion rate and thermal properties of the catalytic reaction. As expected, different metals and their hybrids have different reaction activities. So, the Cu/Pd-PNIPAM was found to have the highest catalytic activities as compare to PNIPAM-Cu and PNIPAM-Pd. The improved catalytic efficiency of bimetallic Cu/Pd nanocomposites for the reduction of methylene blue was observed as compared to the recently published literature (Table 2).

**Table 2:** Comparison of the PNIPAM-Cu/Pd catalyst with the literature reported catalysts for the MB reduction.

Catalyst	Kapp (min^−1^)	References
Ag-NP	0.182	^59^
p(EP-g-AA)-Cu	0.04151	^60^
QAMPS/VP-Fe_3_O_4_ QAMPS	0.0032	^61^
Fe_3_O_4_ QAMPS/VP-Ag	0.0053	^61^
PNIPAM-Cu/Pd	0.173	**This work**

**Figure 10:**
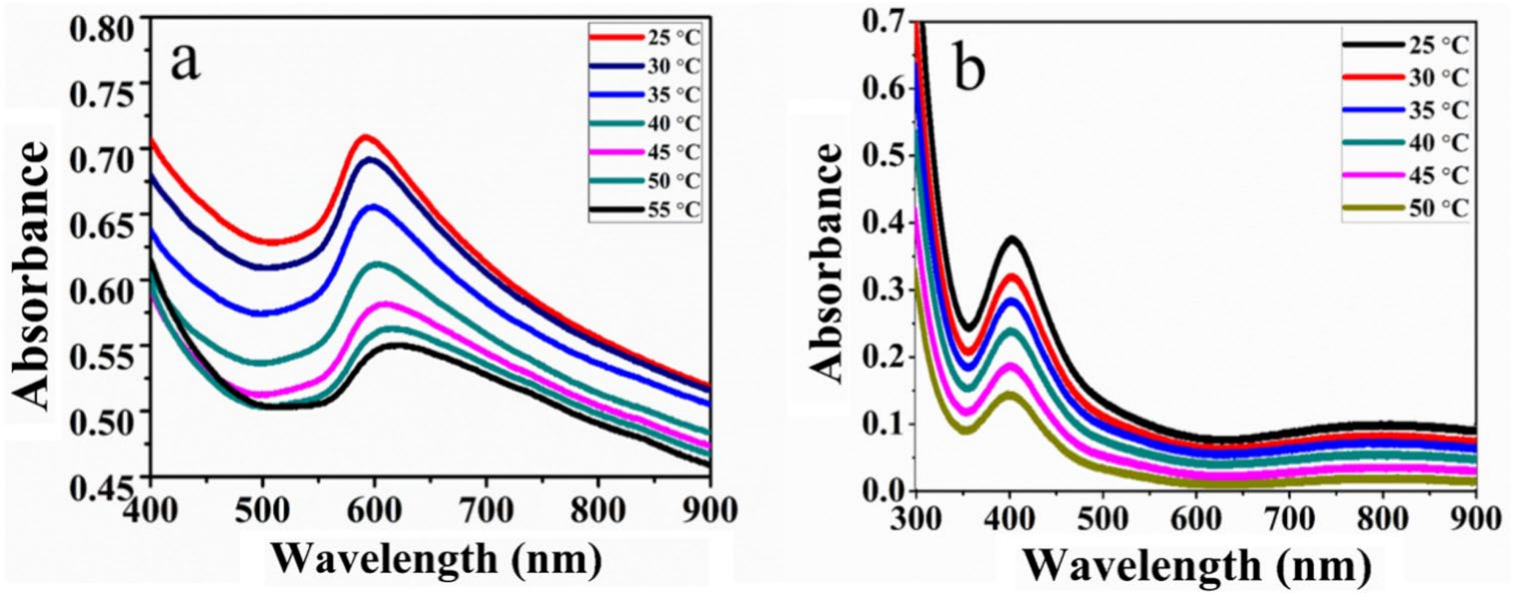
(**a**) Effect of temperature on the UV–Vis spectra of PNIPAM-Cu hybrid microgels and (**b**) PNIPAM-Cu/Pd hybrid microgels.

In-situ the metallic nanoparticles Cu, Pd and Cu/Pd nanoparticles were synthesized in PNIPAM microgels by the reduction of metals salts precursors in the presence of thiol-functionalized microgels and hydrazine hydrate. The localized surface resonance band was observed at 600 nm for copper hybrid microgels. Increase in temperature decreases the size and inter particle distance which increases the refractive index and causes red shift of local resonance band for Cu Nanoparticles as shown in Fig. 10a,b. The red shift becomes larger with increasing temperature from 25 to 55 °C, which broadened the LSPR band gradually. Whereas the Plasmon coupling of the approaching particles causes red shifting due to decrease of band intensity.

## Conclusion

By using the soap-free emulsion polymerization, the thiol functionalized PNIPAM microgel was prepared, which was used for the in situ preparation of nanocomposites impregnated with metals such as copper, palladium and copper palladium alloy. The Copper Palladium Alloy nanocomposites have shown excellent catalytic properties by degrading Nitro phenol and methylene blue in the presence of NaBH_4_. This high catalytic performance is due to the hydrogels network which provided stability for metal nanoparticles. SEM and TEM images showed that metal nanoparticles in the microgels network have no aggregation. The PNIPAM microgels with the functionalization of thiol group were obtained by developing an amide bond between AET and carboxyl-functionalized PNIPAM microgel. The main significance of this protocol is that the thiol group and MNPs distribution can be controlled by the carboxyl group within the PNIPAM microgels backbone. It is due to the presence of carboxyl containing monomers having specific reactivity ratios.

## Supplementary Information

Supplementary Figures.
