# Supplementary material for: Synthesis of bimetallic nanoparticles loaded on to PNIPAM hybrid microgel and their catalytic activity
Source: Sci Rep. 2021 Jul 20;11:14759. doi: 10.1038/s41598-021-94177-6 (PMC8292321; doi:10.1038/s41598-021-94177-6)
Supplement: Supplementary file 1 — Supplementary Figures. [file 41598_2021_94177_MOESM1_ESM.docx]

**Supplementary data**

**Synthesis of bimetallic nanoparticles loaded on to PNIPAM hybrid microgel and their catalytic activity.**

**Running Title:** Synthesis and characterization of hybrid microgels

***Mohib Ullah Kakar^a,b^, Khakemin khan^c^, Muhammad Akram^d^ ,* Rokayya Sami^e^*∗*, *Ebtihal Khojah*^e^ , Imran Iqbal^f^, MahmoudHelal^g^, *Abdul Hakeem^b^, YulinDeng^a^, Rongji Dai^a^∗***

*^a^Beijing Key Laboratory for Separation and Analysis in Biomedicine and Pharmaceutical, Beijing Institute of Technology, (BIT), Beijing 100081, P. R. China;*

*^b^Faculty of Marine Sciences, Lasbela University of Agriculture, Water and Marine Sciences, (LUAWMS), Uthal, Balochistan, Pakistan*

*^c^Department of chemistry,Hazara University Khyber pakthtoonkhawa,Pakistan*

*^d^Institute for Synthetic Biosystem, School of Chemistry and Chemical Engineering, Beijing Institute of Technology, Beijing 100081, China*

^e^ *Department of Food Science and Nutrition, College of Sciences, Taif University, P.O. 11099, Taif 21944, Saudi Arabia.*

*^f^Department of Information and Computational Sciences, School of Mathematical Sciences and LMAM, Peking University, Beijing, 100871, China*

*^g^Department of Mechanical Engineering ,Faculty of Engineering,Taif University, Saudi Arabia*

**Corresponding Author at:*Rokayya Sami,*Department of Food Science and Nutrition, College of Sciences, Taif University, P.O. 11099, Taif 21944, Saudi Arabia.*

*E-mail address:*rokayya@yahoo.com

**Corresponding Author at: Rongji Dai, Beijing Key Laboratory for Separation and Analysis in Biomedicine and Pharmaceutical, Beijing Institute of Technology (BIT), Beijing 100081, P. R. China; E-mail address:* [*dairongji@bit.edu.cn*](mailto:dairongji@bit.edu.cn) *(R. Dai).*

**Supplementary data**

**

**

**Fig. S1 The rate of reduction of 4-Nitrophenol by PNIPAM/Pd hybrid microgels and PNIPAM/Cu hybrid microgels**

**

**

**Fig.S2 ln(C_t_/C_0_) were calculated and plotted as a function of reaction time**

**
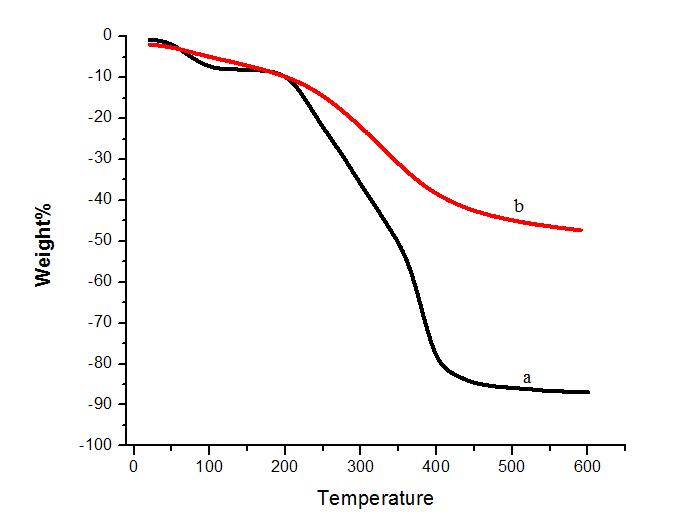
**

**Figure S3 TGA curves for (a) thiol-functionalized PNIPAM microgels, and (b) PNIPAM/Cu hybrid microgels**
